# Empirical Bayesian analysis of paired high-throughput sequencing data with a beta-binomial distribution

**DOI:** 10.1186/1471-2105-14-135

**Published:** 2013-04-23

**Authors:** Thomas J Hardcastle, Krystyna A Kelly

**Affiliations:** 1 Department of Plant Sciences, University of Cambridge, Downing Street, Cambridge, CB2 3EA, UK

## Abstract

**Background:**

Pairing of samples arises naturally in many genomic experiments; for example, gene expression in tumour and normal tissue from the same patients. Methods for analysing high-throughput sequencing data from such experiments are required to identify differential expression, both within paired samples and between pairs under different experimental conditions.

**Results:**

We develop an empirical Bayesian method based on the beta-binomial distribution to model paired data from high-throughput sequencing experiments. We examine the performance of this method on simulated and real data in a variety of scenarios. Our methods are implemented as part of the RbaySeq package (versions 1.11.6 and greater) available from Bioconductor (http://www.bioconductor.org).

**Conclusions:**

We compare our approach to alternatives based on generalised linear modelling approaches and show that our method offers significant gains in performance on simulated data. In testing on real data from oral squamous cell carcinoma patients, we discover greater enrichment of previously identified head and neck squamous cell carcinoma associated gene sets than has previously been achieved through a generalised linear modelling approach, suggesting that similar gains in performance may be found in real data. Our methods thus show real and substantial improvements in analyses of high-throughput sequencing data from paired samples.

## Background

High-throughput sequencing technologies [1-4] allow the measurement of expression of multiple genomic loci in terms of discrete *counts*. A number of methods have been developed in recent years for the detection of differential expression in high-throughput sequencing data. The data are generally modelled using an over-dispersed Poisson distribution (generally the negative-binomial distribution [5-7]), although the beta-binomial distribution [8] has also been used. These methods offer relatively robust and sensitive detection of differential expression either through pairwise comparisons [6,7] or a model-based approach [5].

Analysis methods for an important class of experimental design, that involving paired data, are less well developed. In a paired experimental design, we are generally interested in examining how the ratio of expression between paired counts varies, a scenario that arises naturally in a number of important settings. For example, in oncological studies we may take normal and tumour tissue from the same patient and wish to determine whether the ratio of gene expression differs from a one-to-one ratio between patients within a treatment group, or whether this ratio varies between treatment groups. Similarly, we may wish to compare individuals pre- and post-infection to establish how different strains of a species respond to infection. Paired samples provide a useful approach to such problems as even when the expression of particular genes varies substantially between individuals, the effect of treatment may be relatively consistent. By using paired samples, we can account for individual-specific effects and consequently better detect treatment effects.

Two key questions arise in analyses of paired data. Firstly, we can examine differential expression *within* each pair. That is, we are interested in distinguishing those data which show an approximately one-to-one ratio of expression (after appropriate normalisation) for each pair of counts, and those which show a consistent change between each pair. In the examples above, this is equivalent to discovering differential expression between normal and tumour tissue, or between pre- and post-infection cases, taking into account individual-specific effects. In the second case, we are interested in discovering differential expression *between* groups of paired samples. In our examples, this would correspond to changes in relative expression as a result of treatment. Depending on the nature of the experiment and the data produced, either or both of these forms of differential expression may be of interest.

We present here an empirical Bayesian method based on an over-dispersed binomial distribution, the beta-binomial, for addressing the problem of detecting both types of differential expression in paired sequencing data. The beta-binomial distribution has previously been suggested as a suitable model for the analysis of unpaired high-throughput sequencing data [8], in which the number of reads observed at a single genomic locus is modelled as a proportion of the total number of reads sequenced. In contrast, we model the number of reads observed at a single genomic locus in one member of a pair of samples as a proportion of the number of reads observed at that locus in both samples. Consequently, the application and interpretation of the methods we develop here are substantially different from those of previous work in the analysis of high-throughput sequencing data.

Analyses that account for paired data have thus far employed simplifying assumptions that neglect the full structure of the data. The only published method that has attempted the analysis of paired data is the generalised linear model approach implemented in the edgeR Bioconductor package and described in McCarthy *et al*[9]. We refer to this method subsequently as the edgeR-GLM method. However, this method assumes a log-linear model for the data. This approximation may be appropriate for highly expressed genomic loci, but is likely to lack precision for lowly expressed genomic loci, in which the discrete nature of count data is particularly pertinent to their analysis. A similar generalised linear model approach is implemented in the DESeq Bioconductor package [7], which we refer to subsequently as the DESeq-GLM method. We compare these alternatives to the approach developed here, and show that our approach offers gains in performance on both simulated and real data.

## Methods

The data from high-throughput sequencing experiments used in differential expression analysis may be thought of as a set of *tags*, defining the unique reads sequenced in the experiment, and a set of *counts*, giving the number of times each tag is observed in each of the sequenced libraries made from the samples. In many cases, the data for individual tags is combined to give a count for a larger genomic object. A common example is the summation of tags that map to a gene to give a single value for that gene’s expression. However, the same methods apply to any genomic object whose ‘expression’ can be quantified by high-throughput sequencing, whether that object is a single tag, a gene, miRNA, siRNA, methylation locus, *et cetera*. For each distinct genomic object, we thus have an ordered list, or *tuple*, of discrete counts with the sample order being identical in each tuple.

In analyses of paired data, we introduce the concept of a *tuple pair*. Suppose that we have the count data from a set of *n* samples $A=\{A_{1},\cdots \;,A_{n}\}$, paired with the samples $A^{\prime }=\{A_{1}^{\prime },\cdots \;,A_{n}^{\prime }\}$ respectively so that samples *A*_*i*_ and $A_{i}^{\prime }$ form a *sample pair*. We define the observed data for a particular tuple pair, *c*, as (*u*_1*c*_, ⋯, *u*_*nc*_) where *u*_*ic*_ is the count of the *c*th tuple for sample *A*_*i*_, and the data for the sample pairs as $\left(u_{1c}^{\prime },\cdots \;,u_{\text{nc}}^{\prime }\right)$ where $u_{\text{ic}}^{\prime }$ is the count of the *c*th tuple for sample $A_{i}^{\prime }$. The data for the tuple pair can then be defined as $D_{c}=\{(u_{1c},\cdots u_{\text{nc}}),(u_{1c}^{\prime },\cdots \;,u_{\text{nc}}^{\prime })\}$. We adapt the methods developed for differential expression analysis in our previous work [5] as these have been reported to show the best performance [10,11] in analysis of high-throughput sequencing data. An empirical Bayesian approach is used to estimate the posterior probabilities of each of a set of models that define patterns of differential expression for each tuple pair.

### Model definitions

In forming a set of models for the data, we consider which patterns are biologically likely. In the simple case of a pairwise comparison, we have count data for some sample pairs from condition *A* and condition *B*. If we suppose that we have two biological replicates for each condition, then there are counts from four sequencing libraries *A*_1_, *A*_2_, *B*_1_, *B*_2_ paired with, respectively, counts from sequencing libraries $A_{1}^{\prime },A_{2}^{\prime },B_{1}^{\prime },B_{2}^{\prime }$. In most cases, it is reasonable to suppose that at least some of the tuple pairs may be unaffected by our experimental conditions *A* and *B*. The count data for the sample pairs in these tuple pairs will then share the same underlying parameters. However, some of the tuple pairs may be influenced by the different experimental conditions *A* and *B*. For such a tuple pair, the data from the sample pairs $\left(A_{1},A_{1}^{\prime }\right)$ and $\left(A_{2},A_{2}^{\prime }\right)$will share a set of underlying parameters, the data from the sample pairs $\left(B_{1},B_{1}^{\prime }\right)$and $\left(B_{2},B_{2}^{\prime }\right)$ will share a set of underlying parameters, but, crucially, these sets of parameters will not be identical.

We can represent the models described in terms of the sets of samples for which the data are equivalently distributed under the model. Thus, the model of no differential expression between experimental conditions can be represented by a single set

$$\left\{(A_{1},A_{1}^{\prime }),(A_{2},A_{2}^{\prime }),(B_{1},B_{1}^{\prime }),(B_{2},B_{2}^{\prime })\right\}$$

The model for differential expression between the two experimental conditions can similarly be represented by the two sets

$$\left\{(A_{1},A_{1}^{\prime }),(A_{2},A_{2}^{\prime })\},\{(B_{1},B_{1}^{\prime }),(B_{2},B_{2}^{\prime })\right\}$$

This set based description of the models allows great flexibility in constructing multiple models that may describe the observed data. The evaluation of the posterior likelihood of such a model based on the observed data for a single tuple pair is described below.

### Posterior likelihood of a model

Consider some model *M* for these data defined by the sets {*E*_1_, ⋯, *E*_*m*_}. If, in this model, the *i*th and *j*th sample pairs $\left(A_{i},A_{i}^{\prime }\right)$and $\left(A_{j},A_{j}^{\prime }\right)$ are in the same set *E*_*q*_, then for these sample pairs, the data at tuple pair *c* shares the same underlying parameters *ζ*_*q*_, and are conditionally independent given these parameters. The *ζ*_*q*_ are in turn drawn from some underlying distribution Θ _*q*_. For computational simplicity, we assume that the *ζ*_*q*_ are independently sampled from the distribution Θ _*q*_ for each set *E*_*q*_.

Given a model *M* for the data, the quantity of interest for each tuple *c* is the posterior likelihood of the model *M* given the data *D*_*c*_, that is

(1) $$P(M|D_{c})\;\;=\;\;\frac{P(D_{c}|M)P(M)}{P(D_{c})}$$

We can then calculate $P(D_{c}|M)$ by considering the marginal likelihood

(2) $$\begin{array}{l} P(D_{c}|M)\;\;=\;\;\underset{q}{\prod }\int_{\zeta_{q}\in \Theta_{q}}\left[\underset{i\in E_{q}}{\prod }P((u_{\text{ic}},u_{\text{ic}}^{\prime })|\zeta_{q})\right]P(\zeta_{q}|\Theta_{q})d\zeta_{q} \end{array}$$

The assumption of independence of the *ζ*_*q*_ reduces the dimensionality of the integral allowing a numerical approximation to this integral to be more easily calculated. We suppose that for each Θ _*q*_ we have a set of values Θ _*q*_ that are sampled from the distribution of Θ _*q*_. Then, following Evans & Swartz [12]

(3) $$P(D_{c}|M)\;\;\approx \;\;\underset{q}{\prod }\frac{1}{\left|\Theta_{q}\right|}\underset{\zeta_{q}\in \Theta_{q}}{\sum }\underset{i\in E_{q}}{\prod }P((u_{\text{ic}},u_{\text{ic}}^{\prime })|\zeta_{q})$$

The task that then remains is to derive the set Θ _*q*_ from the data.

### Beta-binomially distributed data

There are a number of possible distributions which could be used for $(u_{\text{ic}},u_{\text{ic}}^{\prime })|\zeta_{q}$ and *ζ*_*q*_∣Θ _*q*_. We develop our method based on the beta-binomial distribution for the tuple pair data, and derive an empirical distribution for the set of underlying parameters using the whole data set. We justify the use of the beta-binomial through the assumption of a Poisson distribution for the number of sequenced reads for a given tuple *c* from an individual library sequenced for sample *i*. The Poisson distribution has been justified as an approximation to an underlying multinomial distribution [7] and has been shown to be a good approximation for the variation found between technical replicates [13].

If the count *u*_*ic*_ is Poisson distributed, and the count of the paired library $u_{\text{ic}}^{\prime }$ is Poisson distributed, then conditional upon the sum $u_{\text{ic}}+u_{\text{ic}}^{\prime }$, *u*_*ic*_ is binomially distributed with parameter *p* indicating the expected proportion of reads belonging to the first of the sample pairs. However, biological variation will cause this proportion to vary between biological replicates, leading to over-dispersion in the observed data. In the absence of prior knowledge about the nature of this over-dispersion we suggest the beta-binomial model as the most convenient approach to model this over-dispersion.

We suppose that the expected proportion of reads from which *u*_*ic*_ is sampled is *Π*. If the library scaling factors [14,15] of samples *A*_*i*_ and $A_{i}^{\prime }$ are identical, then (ignoring biological variation) this is sufficient to describe the distribution of *u*_*ic*_ and $u_{\text{ic}}^{\prime }$ conditional upon their sum. However, if the library scaling factors *L*_*i*_ and $L_{i}^{\prime }$ are the non-identical library scaling factors of samples *A*_*i*_ and $A_{i}^{\prime }$ respectively, then the expected proportion becomes $p=\frac{\Pi L_{i}}{\Pi L_{i}+(1-\Pi )L_{i}^{\prime }}$.

Using the beta-binomial as a model for over-dispersion, we adopt the following parameterisation for the distribution

$$\begin{array}{lll} P(\{u_{\text{ic}},u_{\text{ic}}^{\prime }\}|\Pi ,\phi ,L_{i},L_{i}^{\prime })\;\; & =\;\;\frac{(u_{\text{ic}}+u_{\text{ic}}^{\prime })!}{u_{\text{ic}}!u_{\text{ic}}^{\prime }!}\; & \;\\ & \;\times \frac{B(u_{\text{ic}}+\alpha ,u_{\text{ic}}^{\prime }+\beta )}{B(\alpha ,\beta )},\phi >0\; & \;\\ \;P(\{u_{\text{ic}},u_{\text{ic}}^{\prime }\}|\Pi ,\phi ,L_{i},L_{i}^{\prime })\;\; & =\;\;\frac{(u_{\text{ic}}+u_{\text{ic}}^{\prime })!}{u_{\text{ic}}!u_{\text{ic}}^{\prime }!}p^{u_{\text{ic}}}{\left(1-p\right)}^{u_{\text{ic}}^{\prime }},\phi =0\; & \; \end{array}$$

where $\alpha =p\frac{1-\phi }{\phi }$ and $\beta =(1-p)\frac{1-\phi }{\phi }$. *Π* defines the expected proportion of reads in *u*_*ic*_ and *ϕ* ∈[0, 1] is a measure of the over-dispersion of the data, where *ϕ* = 0 makes the model equivalent to the binomial distribution.

The variance of the binomial distribution under this parametrisation is $\left(u_{\text{ic}}+u_{\text{ic}}^{\prime })p(1-p\right)$. The variance of the beta-binomial distribution is $\left(u_{\text{ic}}+u_{\text{ic}}^{\prime })p(1-p)(1+(u_{\text{ic}}+u_{\text{ic}}^{\prime }-1)\phi \right)$, making the additional variance in the beta-binomial distribution scale linearly with the dispersion parameter *ϕ* for fixed $u_{\text{ic}}+u_{\text{ic}}^{\prime }$ and *p*.

### Empirically derived distributions

We can derive an empirical distribution for the parameters of a model *M* by sampling from the dataset. For each set of samples *E*_*q*_, we would like to find an estimate of the mean and dispersion of the distribution underlying the data from a single tuple pair; *D*_*c*_. By finding estimates of the mean and dispersion for a large number of tuple pairs, we create the sampling Θ _*q*_. The chief difficulty here lies in properly estimating the dispersion. Suppose that the data from a given tuple pair shows genuine differential expression. If the model that we are testing assumes that there is no differential expression, then the dispersion will be substantially over-estimated for this tuple pair. Since we do not know in advance which tuple pairs are genuinely differentially expressed and which are not, we need to consider the replicate structure of the data in order to properly estimate the dispersions. We define the replicate structure by considering the sets {*F*_1_, ⋯*F*_*s*_} where *i*, *j* ∈ *F*_*r*_ if and only if sample pair $\left(A_{j},A_{j}^{\prime }\right)$is a replicate of sample pair $\left(A_{i},A_{i}^{\prime }\right)$.

Given this structure for the data, we can estimate the dispersion of the data in a tuple pair *D*_*c*_ by maximum-likelihood methods. We consider the likelihood of the tuple pair *D*_*c*_ under the replicate structure to be

(4) $$\begin{array}{c} P(D_{c}|\{F_{1},\cdots F_{s}\})\;\;=\;\;\underset{r\in 1:s}{\prod }\underset{i\in F_{r}}{\prod }P(\{u_{\text{ic}},u_{\text{ic}}^{\prime }\}|\Pi_{\text{rc}},\phi_{c}) \end{array}$$

and choose *Π*_*rc*_ and *ϕ*_*c*_ to maximise this likelihood. This gives us a value for *ϕ*_*c*_, the dispersion of the *c*th tuple pair.

In analysis of paired data, one question of interest may be to identify tuple pairs which show a particular ratio of expression between the sample pairs. The most usual case will be a one-to-one ratio (after accounting for variation in library scaling factor), indicating that there is no differential expression of the tuple pair between the sample pairs. To model this, we simply set the *Π*_*qc*_ as the constant proportion of expression to be examined for all *c*.

Alternatively, we may wish to consider a model in which we are not interested primarily in the value of the ratios of expression between sample pairs, but only on whether these ratios are similar or different amongst various experimental groups defined by the sets *E*_*q*_. To approximate a distribution on the Θ _*q*_ for such a model, we can estimate the proportion *Π*_*qc*_ of reads in the first count of each pair of samples for the tuple pair *c*. We achieve this by using the value previously acquired for *ϕ*_*c*_ and estimating *Π*_*qc*_ by maximum likelihood methods. For notational simplicity, we define the data associated with the set *E*_*q*_ as $D_{\text{qc}}=\{(u_{\text{ic}},u_{\text{ic}}^{\prime }):i\in E_{q}\}$ and consider the likelihood of the tuple pair *D*_*qc*_ to be

$$\begin{array}{c} P(D_{\text{qc}}|\phi_{c})\;\;=\;\;\underset{i\in E_{q}}{\prod }P(\{u_{\text{ic}},u_{\text{ic}}^{\prime }\}|\Pi_{\text{qc}},\phi_{c}) \end{array}$$

We then choose *Π*_*qc*_ to maximise this likelihood for each *q*. We can then form the set Θ _*q*_ = {(*Π*_*qc*_, *ϕ*_*c*_)} by repeating one of these processes for multiple sampled tuple pairs. We can then calculate $P(D_{c}|M)$ from Eqn. 3.

This method of estimating the dispersion assumes that the dispersion of a tuple pair is constant across experimental groups. Where the number of samples is small, this is likely to be the best approach. Where there is an expectation that the dispersion will be substantially different between experimental groups, and there are adequate numbers of replicates, there may be advantages to estimating the dispersions individually for each of the different sets of samples in each model, while still considering the replicate structure within these sets. This is easily done by restricting the data (and corresponding replicate structure) to *D*_*qc*_ when estimating the dispersion in Eqn 4.

### Estimation of prior probabilities of each model

A number of options are available when considering the prior probabilities of each model P(M) required in Eqn 1. If we can estimate these from other sources, this may provide an easy solution. However, in many cases we may not be able to provide a reasonable estimate of prior probabilities. One option is to use the iterative re-estimation of the prior likelihoods as described in our previous work [5]. An alternative approach, which we have found subsequently to give more accurate estimates of the prior probabilities in most cases (data not shown) is to use the Bayesian Information Criterion (BIC). For each tuple pair *D*_*c*_ we apply the BIC to select the most likely model based on the calculated likelihoods *P*(*D*_*c*_|*M*) for each model *M*. This allows us to estimate the proportion of data that are best modelled by each of the models, which can be used as an estimate of the prior probablities of each model when calculating *P*(*M*|*D*_*c*_) for any individual tuple pair *D*_*c*_.

### The scaling factor $P(D_{c})$

Finally, we need to consider the scaling factor $P(D_{c})$ in Eqn. 1. Since the number of possible models is finite, though potentially large, the scaling factor $P(D_{c})$ can be determined by summing $P(D_{c}|M)P(M)$ over all possible *M*. In practice, the number of models may be further reduced by considering only those that are biologically plausible.

### False discovery rates from posterior likelihoods

False discovery rates can be estimated directly from the posterior likelihoods estimated for each model. If the likelihood of a model *M* given the observed data for tuple pair *c* is $p_{c}^{M}$ then the likelihood that this is not the true model for the data is $1-p_{c}^{M}$. If *H*_*m*_ is the set of the top *m* tuple pairs for the given model *M*, the false discovery rate is thus estimable as $\frac{\underset{c\in H_{m}}{\sum }(1-p_{c}^{M})}{\left|H_{m}\right|}$.

## Results and discussion

We use both simulated and real data to compare the beta-binomial method described to the edgeR-GLM [9] and DESeq-GLM methods.

### Simulated data

We base our simulations on those described by Robinson & Smyth [6], simulating ten thousand tuple pairs from *n* sample pairs (giving 2*n* libraries in total). We begin by simulating differential expression within pairings only, that is, some of the tuples are simulated so that the relationship between the paired counts is not one-to-one. A more complex experimental design is then simulated by the inclusion of simulated data in which the ratio of expression between the paired counts also differs between experimental groups.

We assess the performance of the methods by ranking the tuple pairs by their strength of association with each type of differential expression and computing the true and false positive rates using these ranked lists. For increased robustness, we estimate the mean of these rates over one hundred simulations under each set of conditions.

For the *i*th sample of a non-differentially expressed *c*th tuple pair, the paired counts *u*_*ic*_ and $u_{\text{ic}}^{\prime }$ are simulated from Poisson distributions with means *λ*_*c*_*L*_*i*_*Q*_*ic*_*M*_*ic*_ and $\lambda_{c}L_{i}^{\prime }Q_{\text{ic}}(1-M_{\text{ic}})$ respectively. The *λ*_*c*_, which define a baseline of expression for the tuple pair when scaled by the library size, are sampled randomly from a set of values empirically estimated by the edgeR method [6] from a SAGE dataset consisting of both normal and cancerous cells [16]. The *L*_*i*_ and $L_{i}^{\prime }$, representing library scaling factors specific to each sequencing library, are sampled from a uniform distribution between 30000 and 90000.

We simulate individual effects in the data by allowing *Q*_*ic*_ to vary for each sample pair *i* as well as for each tuple pair *c*. We simulate this variation by setting $Q_{\text{ic}}=2^{\nu_{\text{ic}}}$ where *ν* is sampled from a uniform distribution between -2 and 2, allowing for up to sixteen-fold variation in expression between sample pairs due to individual effects.

The *M*_*ic*_ allow us to introduce differences between experimental groups of sample pairs and between the members of a pair, while allowing for variation between biological replicates. They are sampled from a beta distribution with shape parameters $a_{\text{ic}}=\frac{\mu_{\text{ic}}}{\mu_{\text{ic}}+\mu_{\text{ic}}^{\prime }}\frac{1-\phi_{c}}{\phi_{c}}$ and $b_{\text{ic}}=\frac{\mu_{\text{ic}}^{\prime }}{\mu_{\text{ic}}+\mu_{\text{ic}}^{\prime }}\frac{1-\phi_{c}}{\phi_{c}}$. In the case of a non-differentially expressed tuple pair *c*, $\mu_{\text{ic}}=\mu_{\text{ic}}^{\prime }=1$ for all *i*. For differentially expressed tuple pairs, we select the values *μ*_*ic*_ and $\mu_{\text{ic}}^{\prime }$ in various ways to simulate different types of differential expression. The values *ϕ*_*c*_, which indicates the level of dispersion (and hence, biological noise) are drawn from a beta distribution with shape parameters 1, 10.

We begin by simulating the simplest case of a paired analysis. In this scenario we are interested only in the differences *within* paired counts, that is, we search for tuple pairs which show evidence for a deviation from a one-to-one expression ratio between the paired counts. We simulate one thousand differentially expressed tuple pairs. For a differentially expressed tuple pair *c*, $\mu_{\text{ic}}=2^{f_{c}}$ and $\mu_{\text{ic}}^{\prime }=2^{-f_{c}}$ for each *i*, where *f*_*c*_ is drawn from a uniform distribution between -*b* and *b*, where *b* is allowed to vary.

**Figure 1:**
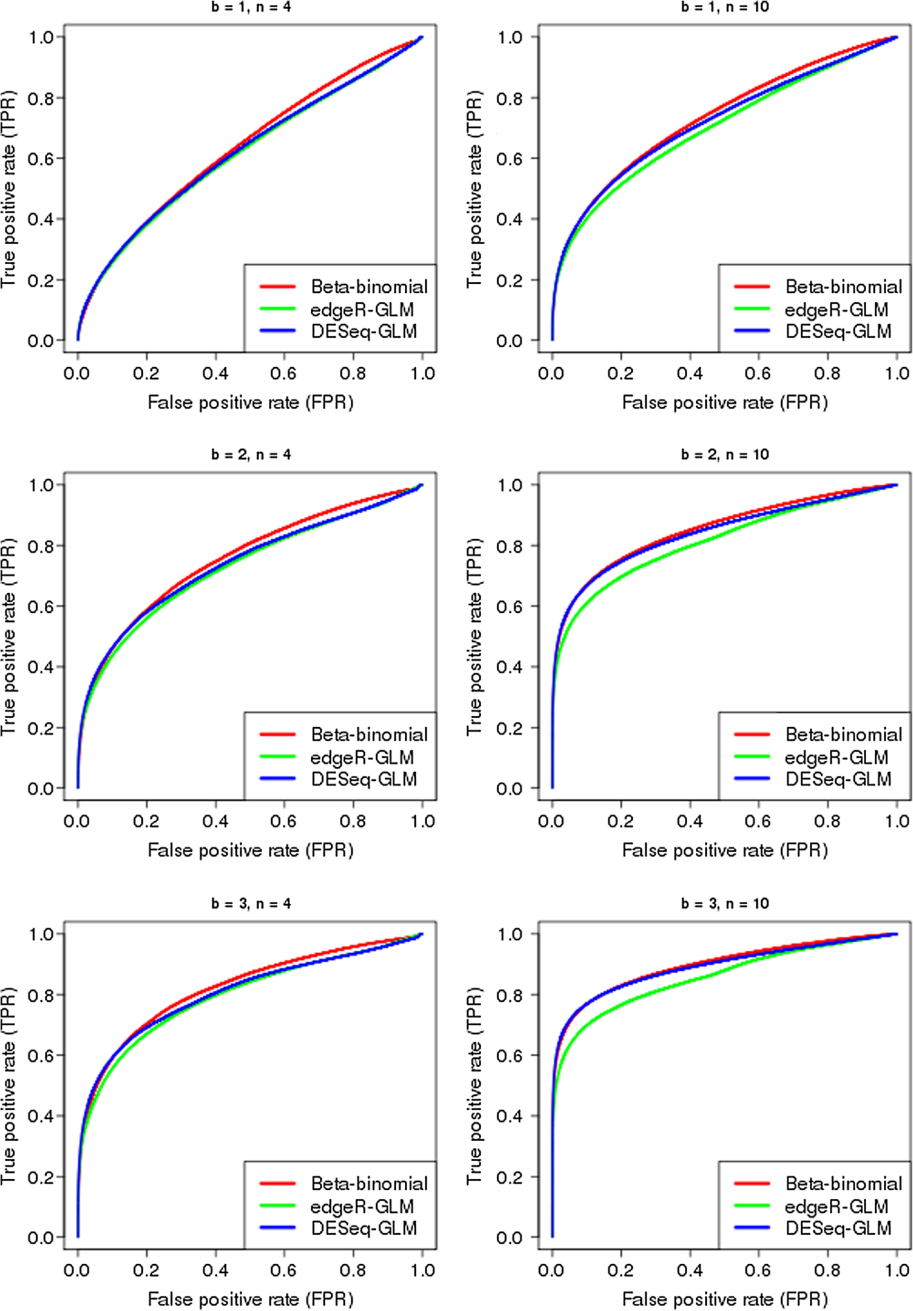
**Comparison of methods identifying differential expression within paired counts.** ROC curves showing the performance of the beta-binomial, edgeR-GLM and DESeq-GLM methods in identifying differential expression within paired counts in simulated data for various combinations of *b*, a measure of the level of differential expression, and *n*, the number of paired libraries.

We examined the performance of the methods on the basis of ROC curves. Figure 1 demonstrates the performance of the methods on simulated data for *b* = 1, 2 and 4 for *n* = 4 and 10.

For low false positive rates, the performance of the methods is approximately equal as each identify the ‘low-hanging fruit’, those tuple pairs showing high differential expression with relatively low biological variation. However, for higher false positive rates the beta-binomial method shows a clear and consistent gain in performance over the generalised linear modelling approaches. The DESeq-GLM in general performs better than edgeR-GLM, especially for higher numbers of sequenced libraries. For high library numbers, the performance of DESeq-GLM approaches that of our beta-binomial approach.

We next consider the more complex case where differential expression exists both within paired counts, and between experimental groups. This is equivalent to an experimental set-up in which we have sample pairs from condition *A*, *A*_1_, …, *A*_*n*_ paired with samples $A_{1}^{\prime },\ldots ,A_{n}^{\prime }$ respectively, and sample pairs from condition *B*, *B*_1_, …, *B*_*n*_ paired with $B_{1}^{\prime },\ldots ,B_{n}^{\prime }$ respectively. We want to find not only tuple pairs that show a consistent variation of expression from a one-to-one ratio within paired counts across all sample pairs (as before), but also those which show an altered ratio of expression between conditions *A* and *B*.

We again simulate ten thousand tuple pairs. For one thousand of these, we simulate differential expression within paired counts as before. For a second group of one thousand tuple pairs, we also simulate differential expression between experimental conditions. We simulate differential expression between experimental conditions by applying a scaling factor *g*_*c*_ to one of the two experimental conditions. This is applied such that for a differentially expressed tuple pair *c*, the data for half the sample pairs, representing the first experimental condition, are simulated using the values $\mu_{\text{ic}}=2^{f_{c}}2^{I_{\text{ic}}g_{c}}$ and $\mu_{\text{ic}}^{\prime }=2^{-f_{c}}2^{-I_{\text{ic}}g_{c}}$. For the remaining half of the sample pairs, representing the second experimental condition, using the values $\mu_{\text{ic}}=2^{f_{c}}2^{(1-I_{\text{ic}})g_{c}}$ and $\mu_{\text{ic}}^{\prime }=2^{-f_{c}}2^{-(1-I_{\text{ic}})g_{c}}$. Here *f*_*c*_ is simulated as before and *g*_*c*_ is drawn from a uniform distribution between -*d* and *d*. *I*_*ic*_ is an indicator variable randomly sampled from {0,1} for each tuple pair *c* indicating whether the effect is assigned to the first or second experimental condition.

Both the beta-binomial approach and the generalised linear modelling approaches are capable of simultaneously detecting both types of differential expression, however, the form of results acquired by these two approaches differs. For the beta-binomial approach, posterior likelihoods are calculated for each available model, and hence only one model for each tuple pair can be assigned a high posterior likelihood. If the true differential expression of a tuple pair involves changes in expression ratios between experimental groups, the model for consistent change from a one-to-one ratio between paired counts will have a low posterior likelihood as the change will not be consistent across the tuple pair. For the generalised linear modelling approaches, both a pair effect and an experimental group effect, and the significance with which these differ from zero, are calculated for each tuple pair. Consequently, both effects can be present with high significance even when changes in expression are driven primarily by a change in expression ratios between experimental groups.

If those tuple pairs simulated as showing differential expression ratios between experimental groups are treated as false positives when considering differences from a one-to-one ratio between paired counts, this heavily penalises the generalised linear model methods. If they are treated as true positives, the generalised linear modelling approaches are evaluated on the basis of two thousand true positives where the beta-binomial method is evaluated on the basis of one thousand true positives, making performance comparisons difficult. To allow fair comparisons between the methods, we therefore exclude the thousand tuple pairs simulated as showing differential expression ratios between experimental groups when calculating the true and false positive rates for detection of differences from a one-to-one ratio within paired counts.

**Figure 2:**
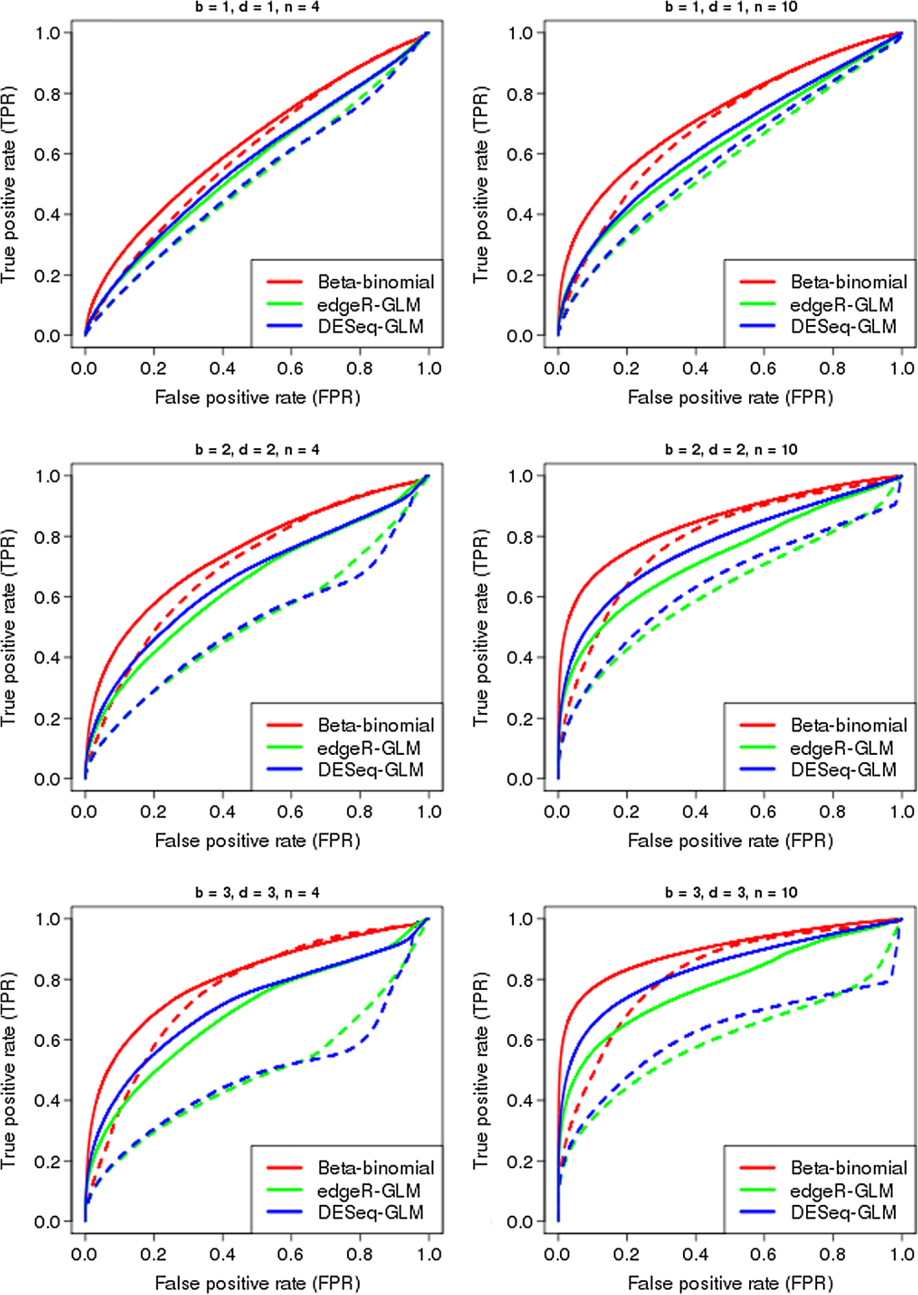
**Comparison of methods identifying differential expression within paired counts and between experimental groups.** ROC curves showing the performance of the methods in simultaneously identifying differences from a one-to-one ratio within paired counts (solid lines) and differential expression ratios between experimental groups (dashed lines) in simulated data for various combinations of *b*, the level of differential expression within paired counts, *d*, the level of differential expresssion between experimental groups, and *n*, the number of paired libraries.

Figure 2 shows the performance of the two methods for the simulation studies as they attempt to discover both differential expression within paired counts and differential expression between experimental groups for a range of values of *b*, *d* and *n*.

In this more complex case, the difference between the performance of the methods is considerably more pronounced. Particularly in identifying differential expression between experimental groups, the beta-binomial method shows considerably better performance than that of both generalised linear modelling approaches. In identifying differential expression from a one-to-one ratio within paired counts, the performance of the beta binomial method is similar to that shown in Figure 1, where this is the only type of differential expression present in the data. However, the performance of the generalised linear modelling approaches is substantially degraded in this more complex scenario.

The simulated data described above are drawn from sets of Poisson distributions whose parameters are a multiple of a random variable drawn from a beta distribution. Therefore, the simulated data have a beta-binomial distribution, the model proposed for the analysis. We can examine the robustness of the model by considering an alternative distribution for the simulations. Since the Poisson distribution is a well established model for the technical effects observed in high-throughput sequencing data [13] we test the robustness of our method by using the minimax distribution [17] as an alternative to the beta distribution for the random variables *M*_*ic*_. The minimax distribution is also a two-parameter distribution on (0,1) with density

$$f(x)=f(x;\hat{\alpha },\hat{\beta })=\hat{\alpha }\hat{\beta }x^{\hat{\alpha }-1}{\left(1-x^{\hat{\alpha }}\right)}^{\hat{\beta }-1}$$

The moments of this distribution are given in terms of the beta function such that

$$E(X^{r})=\hat{\beta }B(1+\frac{1}{\hat{\alpha }},\hat{\beta })$$

Consequently, it is not possible to establish closed-form expressions for the parameters $\hat{\alpha }$ and $\hat{\beta }$ in terms of the desired mean and variance of the random variables *M*_*ic*_, nor is it possible to define a dispersion parameter for this distribution. In order to select parameters for the minimax distributions used to simulate the data, we therefore calculate the parameters for the beta distribution as described above. We then (numerically) calculate the parameters of the minimax distribution such that the mean and variance of each random variable are identical to those which would be used in the case of the beta distribution. This approach has the advantage that, for given parameters of simulation, the results are directly comparable between those data simulated using a beta distribution and those simulated using a minimax distribution.

Results for the application of the three methods to data simulated using the minimax distribution are shown in Additional file 1 (Figures S1 & S2). These results are consistent with those acquired on the simulated data using the beta distribution, suggesting that the methods we propose are reasonably robust to the underlying distribution of the data.

### Biological data

We examine a set of paired data from a recent study of oral squamous cell carcinoma [18]. The study includes three patients with samples taken from tumour and matched normal tissue. As far as possible, we duplicate the analysis conducted by McCarthy *et al*[9] using the edgeR-GLM method to allow comparison with our beta-binomial approach. Our analysis begins with the processed data provided as supplementary material to Tuch *et al*[18]. We map the RefSeq identifiers included in the dataset to gene symbols using the Bioconductor annotation package org.Hs.eg.db (version 2.7.1). We then discard data associated with a RefSeq identifier whose gene symbol is not identified and all but one of any duplicated gene symbol, keeping the data with the greatest number of exons. This results in paired count data for 10529 genes.

We analyse these data to find both genes displaying a consistent fold-change between tumour and normal tissue, and those genes which show heterogeneity in fold-change between the paired counts belonging to the individual patients. The patients are treated as biological replicates for the purposes of dispersion estimation (Eqn. 4) despite the presence of some genes displaying patient-specific effects. In the absence of true biological replicates, this is required in order to carry out a meaningful analysis. We construct a set of models testing both for consistent differential expression between tumour and normal tissue and for differing ratios of expression between individuals. We acquire posterior likelihoods for each of these models of differential expression and hence can evaluate either the likelihood that each gene displays consistent differential expression between normal and tumour samples, or the likelihood that a gene displays differential expression of any kind (by taking the sum of the posterior likelihoods of all models describing differential expression).

We identify 29 genes displaying a consistent ratio of differential expression between tumour and normal samples at a false discovery rate (FDR) of 0.05 (Additional file 1: Table S1). This is considerably lower than the 1276 genes reported by McCarthy *et al*[9] as differentially expressed between tumour and normal tissue, reflecting the premium that our approach places on consistency of expression ratios across the samples. In examining differential expression of any kind, we discover 2605 genes at a false discovery rate of 0.05, indicating the heterogenous nature of the patients. The effect of this heterogeneity can also be seen in an examination of previously reported genes. Of 25 genes reported by Yu *et al*[19] in a systematic review of head and neck squamous cell carcinoma transcriptomics, we find twenty that have differential expression of some kind between normal and tumour samples with an FDR of less than 0.05; of these, however, only two (MAL and LAMC2) show consistent changes in ratio of expression between normal and tumour samples at the same FDR (Additional file 1: Table S2). This pattern is repeated in the nine genes reported as being of particular interest in Tuch *et al*[18]; we find that seven of the nine genes have differential expression of some kind between tumour and normal at an FDR of 0.05 but none show strong evidence for consistent fold-changes in ratio (Additional file 1: Table S3).

Comparisons with the highest-ranked differentially expressed genes discovered by the edgeR-GLM approach show a more consistent picture. Of the reported ten most significant genes from their analysis, five are also selected in our list of the twenty-nine genes showing consistent differential gene expression ratios at an FDR of 0.05, while the remainder still have an estimated likelihood of consistent differential expression greater than 90%. Rank correlation between the gene lists produced by the two methods is 0.59 if the genes are ranked by the likelihood of consistent differential expression but 0.88 if they are ranked by the likelihood of differential expression of any kind.

As in McCarthy *et al*[9], we also demonstrate the biological relevance of the genes we identify by comparisons with the curated gene sets in the MSigDB database [20]. From the twenty-nine genes identified with consistent differential expression, the MSigDB gene sets identified as showing enrichment are predominantly cancer related (Additional file 1: Tables S4 and S5); of these, the top two sets are from two separate studies of head and neck squamous cell carcinomas [21,22]. Comparisons using the 2605 genes identified as showing differential expression of any kind also show a overwhelming preponderance of cancer related gene sets (Additional file 1: Tables S6 and S7) and identify an extremely high proportion of the up and down-regulated genes from Cromer *et al*[21] as well as in various subsets of genes associated with various subclasses of head and neck squamous-cell carcinomas [22] (subgroup E and F) and a set of hypoxia associated genes in head and neck carcinomas [23].

## Conclusions

We have presented here an empirical Bayesian approach to analysing differential expression in paired sample high-throughput sequencing data based on the beta-binomial distribution. The distributions of the parameters of the beta-binomial distribution are estimated by repeated sampling from the observed data, and these distributions are used to estimate posterior likelihoods for each proposed model of expression for each tuple pair. Estimating the distributions of the prior parameters in this way creates a ‘borrowing’ of information across tuple pairs, as the posterior likelihoods for each tuple are calculated using the observed data for all sampled tuple pairs. In analyses with large numbers of outliers, it may be advantageous to ‘squeeze’ [24] the estimated distributions to diminish the effects of these outliers. However, we do not consider this approach here.

Our method is implemented as part of the software package baySeq (versions 1.11.6 and greater). The methods are computationally intensive but readily parallelisable, so that a full analysis of the Tuch *et al*[18] data can be carried out in approximately fifteen minutes on a single machine with eight 2GHz processors.

As with the most successful approaches to analysis of unpaired sequencing data [10,11], our approach for paired data requires no transformation of the data but deals with raw counts directly. This approach should allow for considerably greater accuracy in the detection of differential expression between paired counts. The model-based approach outlined here extends our previous work in the analysis of high-throughput sequencing data [5] and provides great flexibility in the analysis of complex experimental designs, allowing for various types of differential expression in paired data to be simultaneously identified.

A key assumption made in developing this method concerns the nature of the over-dispersion between samples caused by biological variation. In the absence of available data from which to infer the precise nature of the over-dispersion, we have assumed for computational convenience that the beta distribution is a suitable model for the biological variation in ratios of expression between sample pairs and hence that the distribution of the count data may be modelled with the beta-binomial. The beta distribution is remarkably flexible and is thus likely to be capable of accounting for the behaviour of most paired data, although in certain circumstances this assumption may fail. We note, however, that the principles of the empirical Bayesian approach may be applied for any underlying distribution, and so might be adapted to meet this circumstance.

We demonstrate the performance of our methods on both simulated and real data. In analyses of simulated data using a range of parameters, we show considerable gains in performance compared with two implementations of a generalised linear modelling approach, especially when more complex patterns of differential expression are present in the data. The gain in performance using our methods is particularly marked for larger numbers of samples, a result that is likely to be increasingly important as the cost of sequencing experiments declines, allowing larger studies. This gain in performance is also found when the minimax distribution [17] is used in the simulations, suggesting that our methods are reasonably robust to the underlying distributions that may be present in biological systems.

The analysis of the biological data from Tuch *et al*[18] demonstrates that similarly good results can be attained in real-world scenarios. There is a fairly strong correlation between our results and those from the edgeR-GLM approach [9], particularly for the highest ranked genes. This not surprising; those genes showing the largest and most consistent levels of differential expression will be readily identified by any method. However, the analysis of enrichment of those genes identified by the beta-binomial method in curated gene sets from MSigDB identifies not only an overwhelming preponderance of cancer related genes but also those specifically related to head and neck squamous carcinomas. These results compare favourably with those reported in McCarthy *et al*[9], which, although also showing enrichment in primarily cancer-related sets, do not show the same level of association with head and neck squamous carcinoma gene sets. More detailed comparisons using real data are desirable, but at present no well validated data exists on which to make such comparisons.

The comparison of paired mRNA-Seq samples is a major application for our method. However, there are other key applications. In particular, paired data arise naturally in studies of epigenetic markers, such as chromatin and methylation marks, where the prevalance of a particular marker is compared to a baseline measurement for each marker. Our method is, therefore, likely to have wide applicability not only in cancer and other areas of medicine but also in fundamental life science research.

## Competing interests

The authors declare that they have no competing interests.

## Authors’ contributions

TJH designed and implemented the methods described and drafted the manuscript. KAK helped to draft the manuscript. Both authors read and approved the final manuscript.

## Supplementary Material

Additional file 1 **Supplementary Figures and Tables. Figure S1:** ROC curves showing the performance of the beta-binomial, edgeR-GLM and DESeq-GLM methods in identifying differential expression within paired counts in data simulated using the minimax distribution to simulate biological variation. Simulations are carried out for various combinations of *b*, a measure of the level of differential expression, and *n*, the number of paired libraries. **Figure S2:** ROC curves showing the performance of the beta-binomial, edgeR-GLM and DESeq-GLM methods in simultaneously identifying differential expression from a one-to-one ratio within paired counts (solid lines) and differential expression between experimental groups (dashed lines) in data simulated using the minimax distribution to simulate biological variation. Simulations are carried out for various combinations of *b*, a measure of the level of differential expression within paired counts, *d*, the level of differential expression between experimental groups, and *n*, the number of paired libraries. **Table S1:** The top twenty-nine genes (FDR < 0.05) showing consistent ratios within patients of differential expression between normal and tumour samples. **Table S2:** The twenty-five genes identified by Yu *et al* (2008) as being of interest in a systematic review of head and neck squamous cell carcinoma transcripts, as ranked by their likelihood of showing differential expression of any kind in the Tuch *et al* (2010) data. **Table S3:** The nine genes identified by Tuch *et al* (2010) as being of particular interest, as ranked by their likelihood of showing differential expression of any kind. **Table S4:** Gene sets showing enrichment (*p* < 0.01) in the eight up-regulated in tumour genes showing consistent differential expression at FDR > 0.05. Head and neck squamous cell carcinoma gene sets are highlighted. **Table S5:** Gene sets showing enrichment (*p* < 0.01) in the twenty-one down-regulated in tumour genes showing consistent differential expression at FDR > 0.05. Head and neck squamous cell carcinoma gene sets are highlighted. **Table S6:** Gene sets showing enrichment (top fifty) in the 2033 down-regulated in tumour genes showing any differential expression at FDR > 0.05. Head and neck squamous cell carcinoma gene sets are highlighted. **Table S7:** Gene sets showing enrichment (top fifty) in the 572 up-regulated in tumour genes showing any differential expression at FDR > 0.05. Head and neck squamous cell carcinoma gene sets are highlighted. Click here for file
