# Supplementary material for: Empirical Bayesian analysis of paired high-throughput sequencing data with a beta-binomial distribution
Source: BMC Bioinformatics. 2013 Apr 23;14:135. doi: 10.1186/1471-2105-14-135 (PMC3658937; doi:10.1186/1471-2105-14-135)
Supplement: Additional file 1 — Supplementary Figures and Tables. Figure S1: ROC curves showing the performance of the beta-binomial, edgeR-GLM and DESeq-GLM methods in identifying differential expression within paired counts in data simulated using the minimax distribution to simulate biological variation. Simulations are carried out for various combinations of b, a measure of the level of differential expression, and n, the number of paired libraries. Figure S2: ROC curves showing the performance of the beta-binomial, edgeR-GLM and DESeq-GLM methods in simultaneously identifying differential expression from a one-to-one ratio within paired counts (solid lines) and differential expression between experimental groups (dashed lines) in data simulated using the minimax distribution to simulate biological variation. Simulations are carried out for various combinations of b, a measure of the level of differential expression within paired counts, d, the level of differential expression between experimental groups, and n, the number of paired libraries. Table S1: The top twenty-nine genes (FDR < 0.05) showing consistent ratios within patients of differential expression between normal and tumour samples. Table S2: The twenty-five genes identified by Yu et al (2008) as being of interest in a systematic review of head and neck squamous cell carcinoma transcripts, as ranked by their likelihood of showing differential expression of any kind in the Tuch et al (2010) data. Table S3: The nine genes identified by Tuch et al (2010) as being of particular interest, as ranked by their likelihood of showing differential expression of any kind. Table S4: Gene sets showing enrichment (p < 0.01) in the eight up-regulated in tumour genes showing consistent differential expression at FDR > 0.05. Head and neck squamous cell carcinoma gene sets are highlighted. Table S5: Gene sets showing enrichment (p < 0.01) in the twenty-one down-regulated in tumour genes showing consistent differential expression at FDR > 0.05. Head an [file 1471-2105-14-135-S1.pdf]

Supplementary Figure 1: ROC curves showing the performance of the beta-binomial, edgeR-GLM and DESeq-GLM methods in identifying differential expression within paired counts in data simulated using the minimax distribution to simulate biological variation. Simulations are carried out for various combinations of *b*, a measure of the level of differential expression, and *n*, the number of paired libraries.

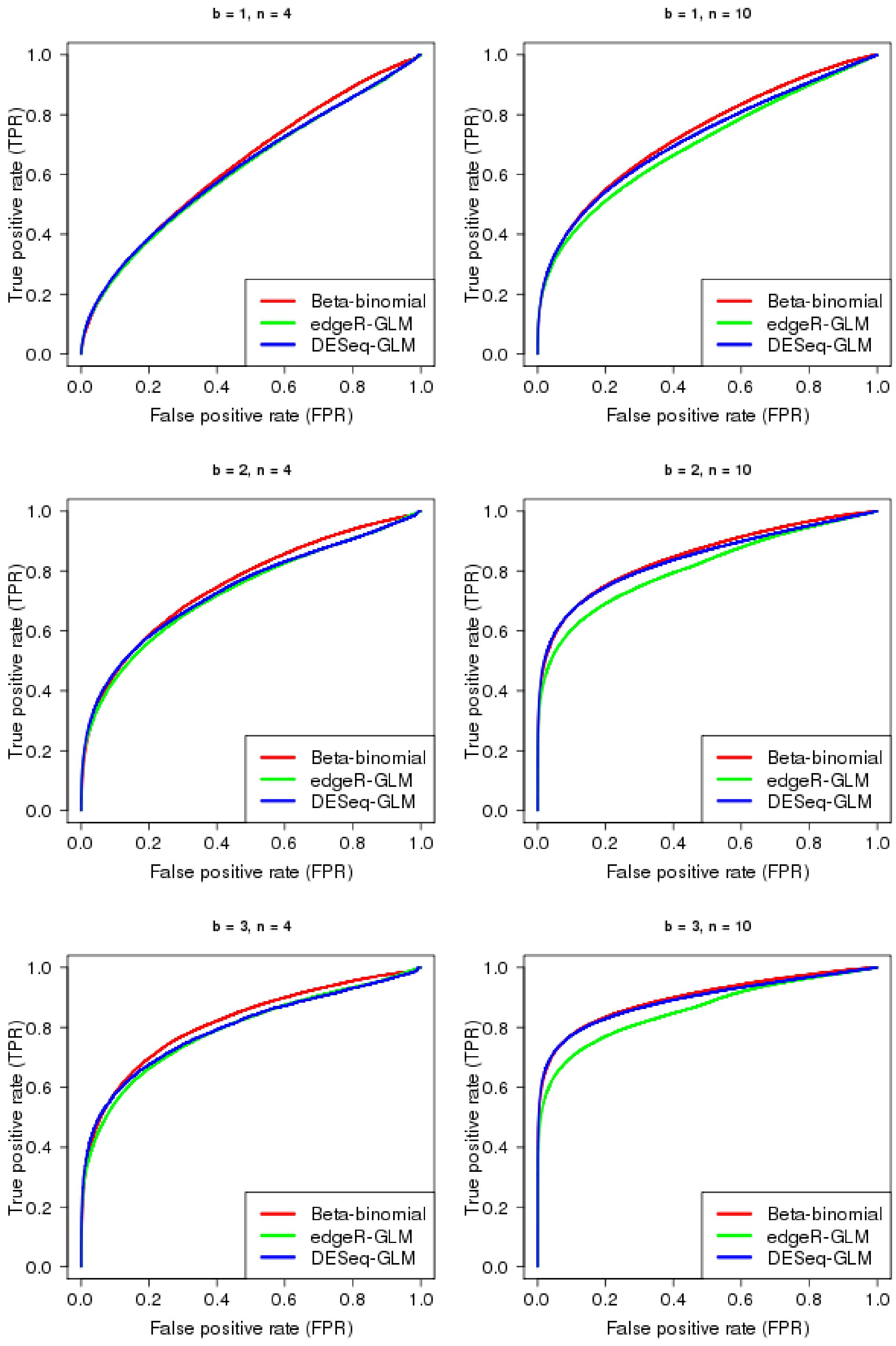

Supplementary Figure 2: ROC curves showing the performance of the beta-binomial, edgeR-GLM and DESeq-GLM methods in simultaneously identifying differential expression from a one-to-one ratio within paired counts (solid lines) and differential expression between experimental groups (dashed lines) in data simulated using the minimax distribution to simulate biological variation. Simulations are carried out for various combinations of *b*, a measure of the level of differential expression within paired counts, *d*, the level of differential expression between experimental groups, and *n*, the number of paired libraries.

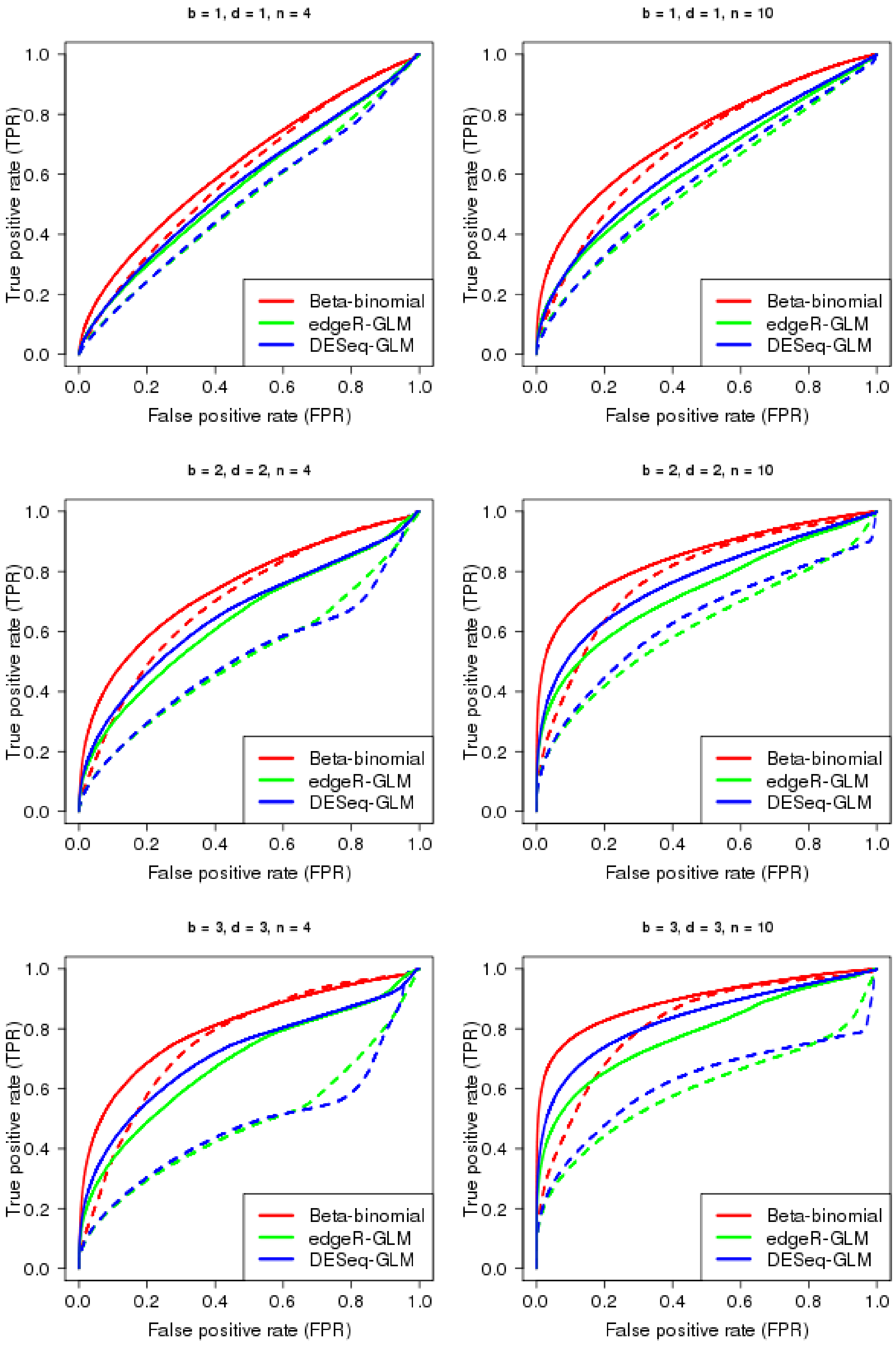

Supplementary Table 1: The top twenty-nine genes (FDR < 0.05) showing consistent ratios within patients of differential expression between normal and tumour samples

RefSeq ID	Gene Symbol	Sample Pair 1 (Normal:Tumour)	Sample Pair 2 (Normal:Tumour)	Sample Pair 3 (Normal:Tumour)	Likelihood of DE	FDR
NM_033641	COL4A6	153:1838	81:1216	58:746	0.990	0.010
NM_021187	CYP4F11	82:1047	21:280	5:82	0.982	0.014
NM_198964	PTHLH	93:1381	147:2323	81:1464	0.975	0.018
NM_182502	TMPRSS11B	3584:5	9457:302	1797:7	0.971	0.020
NM_022440	MAL	2866:2	3559:90	720:5	0.971	0.022
NM_014440	IL36A	507:16	2210:42	427:1	0.971	0.023
NM_018965	TREM2	8:118	8:122	14:150	0.965	0.025
NM_000959	PTGFR	629:11	343:11	924:33	0.965	0.026
NM_006988	ADAMTS1	4142:893	1326:298	1881:423	0.958	0.028
NM_004657	SDPR	295:31	378:45	238:28	0.956	0.030
NM_020247	ADCK3	984:172	653:119	1325:243	0.956	0.031
NM_018891	LAMC2	321:6041	332:3297	571:11045	0.956	0.032
NM_057088	KRT3	1478:11	4573:337	472:4	0.953	0.033
NM_173352	KRT78	4712:272	7462:615	1718:60	0.949	0.034
NM_00111497[illegible]	SMTNL2	365:21	160:7	590:45	0.946	0.036
NM_000602	SERPINE1	174:1387	150:1293	831:6770	0.945	0.037
NM_016190	CRNN	33385:80	26689:2216	6650:19	0.945	0.038
NM_015848	KRT76	8184:43	2305:151	152:4	0.944	0.039
NM_007168	ABCA8	666:41	506:27	938:80	0.944	0.040
NM_00113099[illegible]	RBP1	15:427	16:203	34:377	0.941	0.041
NM_005609	PYGM	1934:36	1535:15	1157:75	0.940	0.042
NM_00112605[illegible]	CASK	521:1926	477:1748	503:1764	0.935	0.043
NM_003042	SLC6A1	568:52	149:20	144:16	0.935	0.044
NM_133646	ZAK	2633:975	2003:760	4150:1605	0.935	0.045
NM_017729	EPS8L1	1092:310	1323:356	424:121	0.931	0.046
NM_001976	ENO3	1510:7	1566:70	2579:162	0.931	0.047
NM_006210	PEG3	207:25	3143:346	652:48	0.929	0.047
NM_015429	ABI3BP	481:3	390:29	1472:76	0.928	0.048
NM_015687	FILIP1	239:16	279:24	662:82	0.926	0.049

Supplementary Table 2: The twenty-five genes identified by Yu *et al* (2008) as being of interest in a systematic review of head and neck squamous cell carcinoma transcripts, as ranked by their likelihood of showing differential expression of any kind in the Tuch *et al* (2010) data.

RefSeq ID	Gene Symbol	Sample Pair 1 (Normal:Tumor)	Sample Pair 2 (Normal:Tumor)	Sample Pair 3 (Normal:Tumor)	Likelihood of DE of any kind	FDR for DE of any kind
NM_022440	MAL	2866:2	3559:90	720:5	0.999	0.001
NM_002272	KRT4	105718:578	120059:19452	25262:23	0.995	0.003
NM_003245	TGM3	35726:2462	28126:4217	6802:46	0.993	0.004
NM_002274	KRT13	544398:3752	435733:99889	112903:1331	0.991	0.005
NM_018891	LAMC2	321:6041	332:3297	571:11045	0.991	0.005
NM_002658	PLAU	339:1042	349:1223	1037:3254	0.989	0.006
NM_001423	EMP1	17130:3756	26831:1815	8999:1634	0.988	0.006
NM_022664	ECM1	3747:287	8415:1104	1199:383	0.986	0.007
NM_002426	MMP12	11:455	35:1008	10:79	0.973	0.013
NM_00104006[illegible]	SPP1	26:599	29:7777	84:1204	0.966	0.016
NM_002425	MMP10	10:123	58:73	153:4445	0.958	0.020
NM_000422	KRT17	3024:28466	3400:8954	6425:16829	0.957	0.021
NM_00113593[illegible]	POSTN	245:1435	614:1217	1713:5714	0.954	0.023
NM_002422	MMP3	61:357	51:511	862:1101	0.953	0.023
NM_000584	IL8	41:693	116:1087	603:553	0.953	0.023
NM_002038	IFI6	611:927	213:367	178:1409	0.947	0.026
NM_002421	MMP1	65:2044	78:1475	4631:15148	0.946	0.027
NM_00107981[illegible]	ITGA6	3251:12060	2404:4156	2395:19845	0.939	0.030
NM_054034	FN1	1002:3125	1008:5527	9071:7292	0.903	0.047
NM_002160	TNC	2996:5901	1875:11903	15073:17141	0.899	0.049
NM_000424	KRT5	130961:95123	118667:75826	48013:137272	0.781	0.102
NM_000393	COL5A2	1800:9135	2543:3381	19188:11021	0.780	0.103
NM_000089	COL1A2	4405:34654	10485:12472	68587:30158	0.776	0.104
NM_001845	COL4A1	2577:13257	4495:4460	22776:7631	0.712	0.135
NM_003118	SPARC	3068:5759	3638:2824	17407:9711	0.614	0.179

Supplementary Table 2: The nine genes identified by Tuch *et al* (2010) as being of particular interest, as ranked by their likelihood of showing differential expression of any kind.

RefSeq ID	Gene Symbol	Sample Pair 1 (Normal:Tumor)	Sample Pair 2 (Normal:Tumor)	Sample Pair 3 (Normal:Tumor)	Likelihood of DE of any kind	FDR for DE of any kind
NM_001231	CASQ1	718:0	1038:3	444:60	0.997	0.003
NM_007191	WIF1	111:67	252:7	40:1	0.984	0.010
NM_002192	INHBA	32:1040	57:978	391:1549	0.949	0.023
NM_002421	MMP1	65:2044	78:1475	4631:15148	0.946	0.031
NM_003483	HMGA2	10:2341	32:377	51:102	0.935	0.038
NM_012309	SHANK2	36:1954	65:216	35:198	0.929	0.043
NM_138565	CTTN	2059:29701	1896:1411	1724:2136	0.920	0.049
NM_053056	CCND1	1903:24957	2431:1758	849:1820	0.875	0.058
NM_078487	CDKN2B	769:5	1000:949	354:3137	0.799	0.074

Supplementary Table 4: Gene sets showing enrichment ($p < 0.01$) in the eight up-regulated in tumour genes showing consistent differential expression at FDR > 0.05. Head and neck squamous cell carcinoma gene sets are highlighted.

Gene Set Name	Description	p value
CROMER_TUMORIGENESIS_UP	***Tumorigenesis markers of head and neck squamous cell carcinoma (HNSCC): up-regulated in the 'early' tumors vs normal samples.***	***3.88E-07***
GU_PDEF_TARGETS_UP	Integrin, VEGF, Wnt and TGF-?? signaling pathway genes up-regulated in PC-3 cells (prostate cancer) after knockdown of PDEF [Gene ID=25803] by RNAi.	1.46E-06
BIOCARTA_PLATELETAPP_PATHWAY	Platelet Amyloid Precursor Protein Pathway	1.00E-05
ONDER_CDH1_TARGETS_2_DN	Genes down-regulated in HMLE cells (immortalized nontransformed mammary epithelium) after E-cadhedrin (CDH1) [Gene ID=999] knockdown by RNAi.	1.26E-05
NGUYEN_NOTCH1_TARGETS_UP	Genes up-regulated in primary keratinocytes by expression of constantly active NOTCH1 [Gene ID=4851].	2.31E-05
JEON_SMAD6_TARGETS_UP	Genes up-regulated in H1299 cells (lung cancer) upon knockdown of SMAD6 [Gene ID=4091] by RNAi.	2.79E-05
MANALO_HYPOXIA_UP	Genes up-regulated in response to both hypoxia and overexpression of an active form of HIF1A [Gene ID=3091].	4.21E-05
KHETCHOUMIAN_TRIM24_TARGETS_UP	Retinoic acid-responsive genes up-regulated in hepatocellular carcinoma (HCC) samples of TRIM24 [Gene ID=8805] knockout mice.	1.19E-04
BROWNE_HCMV_INFECTION_4HR_UP	Genes up-regulated in primary fibroblast cell culture point after infection with HCMV (AD169 strain) at 4 h time point that were not up-regulated at the previous time point, 2 h.	1.57E-04
KEGG_ECM_RECEPTOR_INTERACTION	ECM-receptor interaction	3.80E-04
KEGG_SMALL_CELL_LUNG_CANCER	Small cell lung cancer	3.80E-04
KANG_IMMORTALIZED_BY_TERT_UP	Up-regulated genes in the signature of adipose stromal cells (ADSC) immortalized by forced expression of telomerase (TERT) [Gene ID=7015].	4.26E-04
RICKMAN_HEAD_AND_NECK_CANCER_C	***Cluster c: genes identifying an intrinsic group in head and neck squamous cell carcinoma (HNSCC).***	***6.62E-04***
SWEET_LUNG_CANCER_KRAS_UP	Genes up-regulated in the mouse lung cancer model with mutated KRAS [Gene ID=3845].	7.69E-04
KAN_RESPONSE_TO_ARSENIC_TRIOXIDE	Genes changed in U373-MG cells (malignant glioma) upon treatment with arsenic trioxide [PubChem=14888], a chemical that can cause autophagic cell death.	8.92E-04
PROVENZANI_METASTASIS_DN	Genes down-regulated in polysomal and total RNA samples from SW480 cells (primary colorectal carcinoma, CRC) compared to the SW620 cells (lymph node metastasis from the same individual).	1.09E-03
KIM_WT1_TARGETS_12HR_UP	Genes up-regulated in UB27 cells (osteosarcoma) at 12 hr after inducing the expression of a mutated form of WT1 [Gene ID=7490].	1.49E-03
KIM_WT1_TARGETS_8HR_UP	Genes up-regulated in UB27 cells (osteosarcoma) at 8 hr after inducing the expression of a mutant form of WT1 [Gene ID=7490].	1.51E-03
SMID_BREAST_CANCER_BASAL_DN	Genes down-regulated in basal subtype of breast cancer samles.	1.57E-03
KEGG_FOCAL_ADHESION	Focal adhesion	2.14E-03
KIM_WT1_TARGETS_UP	Genes up-regulated in UB27 cells (osteosarcoma) at any time point after inducing the expression of a mutant form of WT1 [Gene ID=7490].	2.52E-03
KOYAMA_SEMA3B_TARGETS_UP	Genes up-regulated in NCI-H1299 cells (large cell neuroendocrine carcinoma) stably expressing SEMA3B [Gene ID=7869].	2.75E-03
KAAB_HEART_ATRIUM_VS_VENTRICLE_UP	Genes up-regulated in the atria of healthy hearts, compared to venticles.	3.48E-03
JAEGER_METASTASIS_DN	Genes down-regulated in metastases from malignant melanoma compared to the primary tumors.	3.66E-03
CAIRO_HEPATOBLASTOMA_DN	Genes down-regulated in hepatoblastoma samples compared to normal liver tissue.	3.88E-03
BROWNE_HCMV_INFECTION_14HR_DN	Genes down-regulated in primary fibroblast cell culture after infection with HCMV (AD169 strain) at 14 h time point that were not down-regulated at the previous time point, 12 h.	4.70E-03
KEGG_PATHWAYS_IN_CANCER	Pathways in cancer	5.59E-03
SMID_BREAST_CANCER_RELAPSE_IN_BONE_DN	Genes down-regulated in bone relapse of breast cancer.	5.90E-03
DELYS_THYROID_CANCER_UP	Genes up-regulated in papillary thyroid carcinoma (PTC) compared to normal tissue.	8.22E-03
DACOSTA_UV_RESPONSE_VIA_ERCC3_COMMON_DN	Common down-regulated transcripts in fibroblasts expressing either XP/CS or TDD mutant forms of ERCC3 [Gene ID=2071], after UVC irradiation.	9.03E-03
SATO_SILENCED_BY_METHYLATION_IN_PANCREATIC_CANCER_1	Genes up-regulated in the pancreatic cancer cell lines (AsPC1, Hs766T, MiaPaCa2, Panc1) but not in the non-neoplastic cells (HPDE) by decitabine (5-aza-2'-deoxycytidine) [PubChem=451668].	9.96E-03

Supplementary Table 5: Gene sets showing enrichment ($p < 0.01$) in the twenty-one down-regulated in tumour genes showing consistent differential expression at FDR > 0.05. Head and neck squamous cell carcinoma gene sets are highlighted.

Gene Set Name	Description	p value
**RICKMAN_HEAD_AND_NECK_CANCER_E**	**Cluster e: genes identifying an intrinsic group in head and neck squamous cell carcinoma (HNSCC).**	**8.85E-07**
DODD_NASOPHARYNGEAL_CARCINOMA_UP	Genes up-regulated in nasopharyngeal carcinoma (NPC) compared to the normal tissue.	4.91E-04
KUNINGER_IGF1_VS_PDGFB_TARGETS_UP	Genes up-regulated in C2AS12 cells (myoblast) by IGF1 [Gene ID=3479] vs PDGFB [Gene ID=5155].	5.97E-04
**RICKMAN_HEAD_AND_NECK_CANCER_F**	**Cluster f: genes identifying an intrinsic group in head and neck squamous cell carcinoma (HNSCC).**	**9.76E-04**
HELLEBREKERS_SILENCED_DURING_TUMOR_ANGIOGENESIS	Genes down-regulated in tumor-conditioned vs quiescent endothelial cells and up-regulated upon treatment with decitabine and TSA [PubChem=451668, 5562].	1.01E-03
REACTOME_GLUCOSE_METABOLISM	Genes involved in Glucose metabolism	1.01E-03
NING_CHRONIC_OBSTRUCTIVE_PULMONARY_DISEASE_UP	Up-regulated genes in lung tissue of smokers with chronic obstructive pulmonary disease (COPD) vs smokers without disease (GOLD-2 vs GOLD-0).	1.76E-03
LIN_SILENCED_BY_TUMOR_MICROENVIRONMENT	Genes downregulated in MCF10A cells (breast cancer) co-cultured with cancer-associated fibroblasts (CAF).	3.37E-03
VECCHI_GASTRIC_CANCER_EARLY_DN	Down-regulated genes distinguishing between early gastric cancer (EGC) and normal tissue samples.	3.65E-03
CERVERA_SDHB_TARGETS_2	Genes present but differentially expressed between Hep3B cells (hepatocellular carcinoma, HCC) with RNAi knockdown of SDHB [Gene ID=6390] and control cells.	4.04E-03
REACTOME_METABOLISM_OF_CARBOHYDRATES	Genes involved in Metabolism of carbohydrates	4.47E-03
ACEVEDO_METHYLATED_IN_LIVER_CANCER_DN	Genes whose DNA is hyper-methylated in hepatocellular carcinoma (HCC) compared to normal liver.	6.75E-03
ASTON_MAJOR_DEPRESSIVE_DISORDER_DN	Genes down-regulated in the temporal cortex samples from patients with major depressive disorder.	8.22E-03

Supplementary Table 6: Gene sets showing enrichment (top fifty) in the 2033 down-regulated in tumour genes showing any differential expression at FDR > 0.05. Head and neck squamous cell carcinoma gene sets are highlighted.

Gene Set Name	Description	p value
SMID_BREAST_CANCER_LUMINAL_B_DN	Genes down-regulated in the luminal B subtype of breast cancer.	0.00E+00
RODRIGUES_THYROID_CARCINOMA_ANAPLASTIC_DN	Genes down-regulated in anaplastic thyroid carcinoma (ATC) compared to normal thyroid tissue.	0.00E+00
WEST_ADRENOCORTICAL_TUMOR_DN	Down-regulated genes in pediatric adrenocortical tumors (ACT) compared to the normal tissue.	0.00E+00
THUM_SYSTOLIC_HEART_FAILURE_UP	Genes up-regulated in samples with systolic heart failure compared to normal hearts.	0.00E+00
RIGGI_EWING_SARCOMA_PROGENITOR_UP	Genes up-regulated in mesenchymal stem cells (MSC) engineered to express EWS-FLI1 [Gene ID=2130, 2321] fusion protein.	0.00E+00
SWEET_LUNG_CANCER_KRAS_DN	Genes down-regulated in the mouse lung cancer model with mutated KRAS [Gene ID=3845].	0.00E+00
LIU_PROSTATE_CANCER_DN	Genes down-regulated in prostate cancer samples.	0.00E+00
SCHUETZ_BREAST_CANCER_DUCTAL_INVASIVE_UP	Genes up-regulated in invasive ductal carcinoma (IDC) relative to ductal carcinoma in situ (DCIS, non-invasive).	0.00E+00
SMID_BREAST_CANCER_NORMAL_LIKE_UP	Genes up-regulated in the normal-like subtype of breast cancer.	0.00E+00
ONDER_CDH1_TARGETS_2_UP	Genes up-regulated in HMLE cells (immortalized nontransformed mammary epithelium) after E-cadhedrin (CDH1) [Gene ID=999] knockdown by RNAi.	0.00E+00
LINDGREN_BLADDER_CANCER_CLUSTER_2B	Genes specifically up-regulated in Cluster IIb of urothelial cell carcinom (UCC) tumors.	0.00E+00
RIGGI_EWING_SARCOMA_PROGENITOR_DN	Genes down-regulated in mesenchymal stem cells (MSC) engineered to express EWS-FLI1 [Gene ID=2130, 2321] fusion protein.	0.00E+00
DELYS_THYROID_CANCER_DN	Genes down-regulated in papillary thyroid carcinoma (PTC) compared to normal tissue.	0.00E+00
PICCALUGA_ANGIOIMMUNOBLASTIC_LYMPHOMA_UP	Up-regulated genes in angioimmunoblastic lymphoma (AILT) compared to normal T lymphocytes.	0.00E+00
EBAUER_MYOGENIC_TARGETS_OF_PAX3_FOXO1_FUSION	Muscle development genes up-regulated in Rh4 cells (alveolar rhabdomyosarcoma, ARMS) but not in the RD cells (embryonal rhabdomyosarcoma, ERMS) after knockdown of PAX3-FOXO1 [Gene ID=5077, 2308] fusion by RNAi for 72 hr.	0.00E+00
CROMER_TUMORIGENESIS_DN	***Tumorigenesis markers of head and neck squamous cell carcinoma (HNSCC): down-regulated in the 'early' tumors vs normal samples.***	***0.00E+00***
RICKMAN_HEAD_AND_NECK_CANCER_F	***Cluster f: genes identifying an intrinsic group in head and neck squamous cell carcinoma (HNSCC).***	***0.00E+00***
SMID_BREAST_CANCER_BASAL_DN	Genes down-regulated in basal subtype of breast cancer samles.	1.11E-16
ONKEN_UVEAL_MELANOMA_DN	Genes down-regulated in uveal melanoma: class 2 vs class 1 tumors.	1.11E-16
LINDGREN_BLADDER_CANCER_CLUSTER_1_DN	Genes whose expression profile is specific to Custer I of urothelial cell carcinoma (UCC) tumors.	1.11E-16
NUYTTEN_EZH2_TARGETS_UP	Genes up-regulated in PC3 cells (prostate cancer) after knockdown of EZH2 [Gene ID=2146] by RNAi.	4.44E-16
NAKAYAMA_SOFT_TISSUE_TUMORS_PCA2_DN	Top 100 probe sets contrubuting to the negative side of the 2nd principal component; associated with adipocytic differentiation.	4.44E-16
VECCHI_GASTRIC_CANCER_EARLY_DN	Down-regulated genes distinguishing between early gastric cancer (EGC) and normal tissue samples.	5.55E-16
KINSEY_TARGETS_OF_EWSR1_FLII_FUSION_DN	Genes down-regulated in TC71 and EWS502 cells (Ewing's sarcoma) upon knockdown of the EWSR1-FLII fusion [Gene ID=2130, 2314].	9.99E-16
RICKMAN_HEAD_AND_NECK_CANCER_E	***Cluster e: genes identifying an intrinsic group in head and neck squamous cell carcinoma (HNSCC).***	***2.22E-15***
SMID_BREAST_CANCER_LUMINAL_A_UP	Genes up-regulated in the luminal A subtype of breast cancer.	2.22E-15
ENK_UV_RESPONSE_EPIDERMIS_DN	Genes down-regulated in epidermis after to UVB irradiation.	1.32E-14
TURASHVILI_BREAST_DUCTAL_CARCINOMA_VS_DUCTAL_NORMAL_DN	Genes down-regulated in ductal carcinoma vs normal ductal breast cells.	5.51E-14
SANA_TNF_SIGNALING_DN	Genes down-regulated in five primary endothelial cell types (lung, aortic, iliac, dermal, and colon) by TNF [Gene ID=7124].	5.53E-14
REN_ALVEOLAR_RHABDOMYOSARCO	Genes commonly up-regulated in human alveolar rhabdomyosarcoma	6.02E-14

MA_UP	(ARMS) and its mouse model overexpressing PAX3-FOXO1 [Gene ID=5077, 2308] fusion.	
RODRIGUES_THYROID_CARCINOMA_POORLY_DIFFERENTIATED_DN	Genes down-regulated in poorly differentiated thyroid carcinoma (PDTC) compared to normal thyroid tissue.	7.15E-14
WINTER_HYPOXIA_DN	***Genes down-regulated in head and neck tumor samples which clustered around known hypoxia genes.***	***8.72E-14***
DODD_NASOPHARYNGEAL_CARCINOMA_UP	Genes up-regulated in nasopharyngeal carcinoma (NPC) compared to the normal tissue.	1.31E-13
IZADPANAH_STEM_CELL_ADIPOSE_VS_BONE_DN	Genes down-regulated in adipose tissue mesenchymal stem cells (ASC) vs bone marrow mesenchymal stem cells (rBMSC)	1.86E-13
SABATES_COLORECTAL_ADENOMA_DN	Genes down-regulated in colorectal adenoma compared to normal mucosa samples.	2.84E-13
LIN_SILENCED_BY_TUMOR_MICROENVIRONMENT	Genes downregulated in MCF10A cells (breast cancer) co-cultured with cancer-associated fibroblasts (CAF).	1.30E-12
EBAUER_TARGETS_OF_PAX3_FOXO1_FUSION_UP	Genes up-regulated in Rh4 cells (alveolar rhabdomyosarcoma, ARMS) after knockdown of the PAX3-FOXO1 [Gene iD=5077, 2308] fusion protein by RNAi for 72 hr.	1.32E-12
SENESE_HDAC1_AND_HDAC2_TARGETS_DN	Genes down-regulated in U2OS cells (osteosarcoma) upon knockdown of both HDAC1 and HDAC2 [Gene ID=3065, 3066] by RNAi.	1.32E-12
GRUETZMANN_PANCREATIC_CANCER_UP	Genes up-regulated in pancreatic ductal adenocarcinoma (PDAC) identified in a meta analysis across four independent studies.	1.91E-12
CAIRO_HEPATOBLASTOMA_CLASSES_DN	Genes down-regulated in robust Cluster 2 (rC2) of hepatoblastoma samples compared to those in the robust Cluster 1 (rC1).	1.92E-12
HORIUCHI_WTAP_TARGETS_UP	Genes up-regulated in primary endothelial cells (HUVEC) after knockdown of WTAP [Gene ID=9589] by RNAi.	3.16E-12
CHARAFE_BREAST_CANCER_LUMINAL_VS_MESENCHYMAL_DN	Genes down-regulated in luminal-like breast cancer cell lines compared to the mesenchymal-like ones.	3.95E-12
HELLER_HDAC_TARGETS_SILENCED_BY_METHYLATION_UP	Genes up-regulated in multiple myeloma (MM) cell lines treated with both decitabine [PubChem=451668] TSA [PubChem=5562].	6.58E-12
REACTOME_MUSCLE_CONTRACTION	Genes involved in Muscle contraction	8.36E-12
WAMUNYOKOLI_OVARIAN_CANCER_LMP_DN	Genes down-regulated in mucinous ovarian carcinoma tumors of low malignant potential (LMP) compared to normal ovarian surface epithelium tissue.	1.48E-11
WU_CELL_MIGRATION	Genes associated with migration rate of 40 human bladder cancer cells.	3.07E-11
GOZGIT_ESR1_TARGETS_DN	Genes down-regulated in TMX2-28 cells (breast cancer) which do not express ESR1 [Gene ID=2099]) compared to the parental MCF7 cells which do.	3.35E-11
REACTOME_HEMOSTASIS	Genes involved in Hemostasis	3.51E-11
REN_ALVEOLAR_RHABDOMYOSARCOMA_DN	Genes commonly down-regulated in human alveolar rhabdomyosarcoma (ARMS) and its mouse model overexpressing PAX3-FOXO1 [Gene ID=5077, 2308] fusion.	3.65E-11
CASORELLI_ACUTE_PROMYELOCYTIC_LEUKEMIA_DN	Genes down-regulated in APL (acute promyeolocytic leukemia) blasts expressing PML-RARA fusion [Gene ID=5371, 5914] compared to normal promyeloblasts.	4.37E-11

Supplementary Table 7: Gene sets showing enrichment (top fifty) in the 572 up-regulated in tumour genes showing any differential expression at FDR > 0.05. Head and neck squamous cell carcinoma gene sets are highlighted.

Gene Set Name	Description	p value
PUJANA_BRCA1_PCC_NETWORK	Genes constituting the BRCA1-PCC network of transcripts whose expression positively correlated (Pearson correlation coefficient, PCC >= 0.4) with that of BRCA1 [Gene ID=672] across a compendium of normal tissues.	0.00E+00
DODD_NASOPHARYNGEAL_CARCINOMA_DN	Genes down-regulated in nasopharyngeal carcinoma (NPC) compared to the normal tissue.	0.00E+00
KINSEY_TARGETS_OF_EWSR1_FLII_FUSION_UP	Genes up-regulated in TC71 and EWS502 cells (Ewing's sarcoma) upon knockdown of the EWSR1-FLII fusion [Gene ID=2130, 2314].	0.00E+00
NUYTTEN_EZH2_TARGETS_DN	Genes down-regulated in PC3 cells (prostate cancer) after knockdown of EZH2 [Gene ID=2146] by RNAi.	0.00E+00
CAIRO_HEPATOBLASTOMA_CLASSES_UP	Genes up-regulated in robust Cluster 2 (rC2) of hepatoblastoma samples compared to those in the robust Cluster 1 (rC1).	0.00E+00
RODRIGUES_THYROID_CARCINOMA_ANAPLASTIC_UP	Genes up-regulated in anaplastic thyroid carcinoma (ATC) compared to normal thyroid tissue.	0.00E+00
BERENJENO_TRANSFORMED_BY_RHOA_UP	Genes up-regulated in NIH3T3 cells (fibroblasts) transformed by expression of contitutively active (Q63L) form of RHOA [Gene ID=387] off plasmid vector.	0.00E+00
SMID_BREAST_CANCER_BASAL_UP	Genes up-regulated in basal subtype of breast cancer samles.	0.00E+00
SHEDDEN_LUNG_CANCER_POOR_SURVIVAL_A6	Cluster 6 of method A: up-regulation of these genes in patients with non???small cell lung cancer (NSCLC) predicts poor survival outcome.	0.00E+00
VECCHI_GASTRIC_CANCER_EARLY_UP	Up-regulated genes distinguishing between early gastric cancer (EGC) and normal tissue samples.	0.00E+00
BASAKI_YBX1_TARGETS_UP	Genes up-regulated in SKOC-3 cells (ovarian cancer) after YB-1 (YBX1) [Gene ID=4904] knockdown by RNAi.	0.00E+00
ONDER_CDH1_TARGETS_2_DN	Genes down-regulated in HMLE cells (immortalized nontransformed mammary epithelium) after E-cadhedrin (CDH1) [Gene ID=999] knockdown by RNAi.	0.00E+00
KOBAYASHI_EGFR_SIGNALING_24HR_DN	Genes down-regulated in H1975 cells (non-small cell lung cancer, NSCLC) resistant to gefitinib [PubChem=123631] after treatment with EGFR inhibitor CL-387785 [PubChem=2776] for 24h.	0.00E+00
SOTIRIOU_BREAST_CANCER_GRADE_1_VS_3_UP	Up-regulated genes whose expression correlated with histologic grade of invasive breast cancer tumors: comparison of grade 1 vs grade 3.	0.00E+00
RUIZ_TNC_TARGETS_DN	Genes down-regulated in T98G cells (glioblastoma) by TNC [Gene ID=3371].	0.00E+00
CHIANG_LIVER_CANCER_SUBCLASS_PROLIFERATION_UP	Top 200 marker genes up-regulated in the 'proliferation' subclass of hepatocellular carcinoma (HCC); characterized by increased proliferation, high levels of serum AFP [Gene ID=174], and chromosomal instability.	0.00E+00
CROONQUIST_IL6_DEPRIVATION_DN	Genes down-regulated in the ANBL-6 cell line (multiple myeloma, MM) after withdrawal of IL6 [Gene ID=3569].	0.00E+00
ROSTY_CERVICAL_CANCER_PROLIFERATION_CLUSTER	The 'Cervical Cancer Proliferation Cluster' (CCPC): genes whose expression in cervical carcinoma positively correlates with that of the HPV E6 and E7 oncogenes; they are also differentially expressed according to disease outcome.	0.00E+00
CROMER_TUMORIGENESIS_UP	***Tumorigenesis markers of head and neck squamous cell carcinoma (HNSCC): up-regulated in the 'early' tumors vs normal samples.***	***0.00E+00***
BENPORATH_CYCLING_GENES	Genes showing cell-cycle stage-specific expression [PMID=12058064].	1.11E-16
ODONNELL_TFRC_TARGETS_DN	Genes dpwn-regulated in P493-6 cells (B lymphocyte) upon knockdown of TFRC [Gene ID=7037] by RNAi.	1.11E-16
LY_AGING_OLD_DN	Genes up-regulated in fibroblasts from old individuals, compared to those from young donors.	1.11E-16
WONG_EMBRYONIC_STEM_CELL_CORE	The 'core ESC-like gene module': genes coordinately up-regulated in a compendium of mouse embryonic stem cells (ESC) which are shared with the human ESC-like module.	2.22E-16
LI_WILMS_TUMOR_VS_FETAL_KIDNEY_1_DN	Genes down-regulated in Wilm's tumor samples compared to fetal kidney.	4.44E-16
NAKAYAMA_SOFT_TISSUE_TUMORS_PCA2_UP	Top 100 probe sets contrubuting to the positive side of the 2nd principal component; associated with adipocytic differentiation.	5.55E-16
RODRIGUES_THYROID_CARCINOMA_POORLY_DIFFERENTIATED_UP	Genes up-regulated in poorly differentiated thyroid carcinoma (PDTC) compared to normal thyroid tissue.	1.11E-15
CROONQUIST_NRAS_SIGNALING_DN	Genes down-regulated in ANBL-6 cell line (multiple myeloma, MM)	1.22E-15

	expressing a constantly active form of NRAS [Gene ID=4893] off a plasmid vector compared to those grown in the presence of IL6 [Gene ID=3569].	
DELYS_THYROID_CANCER_UP	Genes up-regulated in papillary thyroid carcinoma (PTC) compared to normal tissue.	3.00E-15
CHARAFE_BREAST_CANCER_LUMINAL_VS_BASAL_DN	Genes down-regulated in luminal-like breast cancer cell lines compared to the basal-like ones.	1.07E-14
RHODES_UNDIFFERENTIATED_CANCER	Genes commonly up-regulated in undifferentiated cancer relative to well-differentiated cancer, based on the meta-analysis of the OncoMine gene expression database.	1.17E-14
WHITEFORD_PEDIATRIC_CANCER_MARKERS	Differentially expressed genes in a panel of xenografts representing 8 common pediatric tumors compared to the normal tissues.	1.40E-14
BLUM_RESPONSE_TO_SALIRASIB_DN	Selected genes down-regulated in response to the Ras inhibitor salirasib [PubChem=5469318] in a panel of cancer cell lines with constantly active HRAS [Gene ID=3265].	1.75E-14
LINDGREN_BLADDER_CANCER_CLUSTER_3_UP	Genes whose expression profile is specific to Cluster III of urothelial cell carcinoma (UCC) tumors.	1.90E-14
PUJANA_CHEK2_PCC_NETWORK	Genes constituting the CHEK2-PCC network of transcripts whose expression positively correlates (Pearson correlation coefficient, PCC >= 0.4) with that of CHEK2 [Gene ID=11200].	1.98E-14
HORIUCHI_WTAP_TARGETS_DN	Genes down-regulated in primary endothelial cells (HUVEC) after knockdown of WTAP [Gene ID=9589] by RNAi.	2.18E-14
WINTER_HYPOXIA_UP	***Genes up-regulated in head and neck tumor samples which clustered around known hypoxia genes.***	***2.70E-14***
LIAO_METASTASIS	Genes up-regulated in the samples with intrahepatic metastatic hepatocellular carcinoma (HCC) vs primary HCC.	5.34E-14
JAEGER_METASTASIS_DN	Genes down-regulated in metastases from malignant melanoma compared to the primary tumors.	5.87E-14
LY_AGING_MIDDLE_DN	Genes down-regulated in fibroblasts from middle-age individuals, compared to those from the young donors.	8.20E-14
CASORELLI_ACUTE_PROMYELOCYTIC_LEUKEMIA_DN	Genes down-regulated in APL (acute promyeolocytic leukemia) blasts expressing PML-RARA fusion [Gene ID=5371, 5914] compared to normal promyeloblasts.	1.15E-13
KAN_RESPONSE_TO_ARSENIC_TRIOXIDE	Genes changed in U373-MG cells (malignant glioma) upon treatment with arsenic trioxide [PubChem=14888], a chemical that can cause autophagic cell death.	1.36E-13
SHEPARD_BMYB_MORPHOLINO_DN	Human orthologs of genes down-regulated in zebra fish after knockdown of BMYB [Gene ID=4605] by morpholino.	1.72E-13
PUJANA_BRCA2_PCC_NETWORK	Genes constituting the BRCA2-PCC network of transcripts whose expression positively correlated (Pearson correlation coefficient, PCC >= 0.4) with that of BRCA2 [Gene ID=675] across a compendium of normal tissues.	1.88E-13
SENESE_HDAC3_TARGETS_UP	Genes up-regulated in U2OS cells (osteosarcoma) upon knockdown of HDAC3 [Gene ID=8841] by RNAi.	2.63E-13
FOURNIER_ACINAR_DEVELOPMENT_LATE_2	Genes identified by method 2 as coordinately down-regulated late in HMEC cells (mammary epithelium) during acinar development in vitro.	3.88E-13
KANG_DOXORUBICIN_RESISTANCE_UP	Genes differentialy expressed in gastric cancer cell lines: doxorubicin [PubChem=31703] resistant vs sensitive.	5.97E-13
WU_CELL_MIGRATION	Genes associated with migration rate of 40 human bladder cancer cells.	7.93E-13
GRAHAM_CML_DIVIDING_VS_NORMAL_QUIESCENT_UP	Genes up-regulated in quiescent CD34+ [Gene ID=8842] cells isolated from peripheral blood of normal donors compared to the dividing cells from CML (chronic myeloid leukemia) patients.	8.96E-13
SHEPARD_BMYB_TARGETS	Human orthologs of BMYB [Gene ID=4605] target genes in zebra fish, identified as commonly changed in the BMYB loss of function mutant crb ('crush and burn') and after knockdown of BMYB by morpholino.	3.24E-12
WEST_ADRENOCORTICAL_TUMOR_UP	Up-regulated genes in pediatric adrenocortical tumors (ACT) compared to the normal tissue.	7.52E-12
